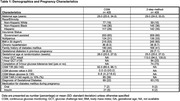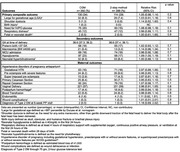# Oral Plenary Session 2 (Fellows Plenary): Friday, January 31, 2025 8:00 AM – 10:00 AM

**DOI:** 10.1002/pmf2.12010

**Published:** 2025-01-05

## 39 | Methylergonovine to Decrease Blood Loss During Cesarean Delivery in Twins: A Triple‐Blinded Placebo‐Controlled Randomized Trial

Helen B. Gomez Slagle^1^; Shai Bejerano^2^; Russell S. Miller^2^; Dena Goffman^2^; Mary E. D'Alton^2^; Mirella Mourad^2^

*^1^Columbia University Irving Medical Center, New York, NY; ^2^Columbia University Medical Center, New York, NY*

8:00 AM ‐ 8:15 AM

**Objective**: To evaluate whether prophylactic administration of intramuscular (IM) methylergonovine after cord clamping reduces blood loss during cesarean delivery (CD) in twin pregnancies.

**Study Design**: This single‐center randomized placebo‐controlled triple‐blinded trial compared the effects on blood loss at CD of IM methylergonovine versus saline placebo control. Pregnant individuals with twin gestations at ^3^34 weeks of gestation undergoing planned CD were enrolled. Methylergonovine or saline placebo was administered immediately after umbilical cord clamping of the second twin in addition to standard care with oxytocin. The primary outcome was change in maternal hemoglobin (Hgb) level from preoperative to postoperative day 1 (POD 1). We planned to enroll 66 patients to detect a standard deviation (SD) greater drop in postoperative maternal Hgb, assuming ‐1.40 (0.83) g/dL Hgb difference in planned CDs (90% power, alpha 0.05). Pearson chi‐square, or Fisher's exact test, and Wilcoxon rank‐sum with intent‐to‐treat principles were performed as appropriate.

**Results**: From February 2023 through March 2024, 77 patients were eligible and 66 participated. There were no demographic differences between methylergonovine and placebo groups. Median gestational age at delivery was 37 weeks for both groups. Mean Hgb drop at POD 1 was 1.1 g/dL (SD 0.7 g/dL) for methylergonovine and 2.1 g/dL (SD 0.9 g/dL) for placebo (p< 0.001). Median quantitative blood loss was significantly lower in patients receiving prophylactic methylergonovine (891cc versus 1017cc, p = 0.003). Postpartum hemorrhage rates were lower in the methylergonovine group (18.2% vs 54.5%, p = 0.002). There were 7 cases of unblinding due to hemorrhage in the placebo group compared to no cases in the methylergonovine group (p = 0.01); unblinded subjects received methylergonovine.

**Conclusion**: Prophylactic IM methylergonovine administered during twin CD after umbilical cord clamping significantly reduced intraoperative blood loss and hemoglobin drop at POD 1. These data support further study of methylergonovine as a preventative treatment strategy at the time of twin CD.

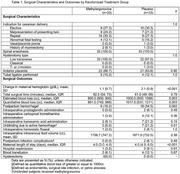

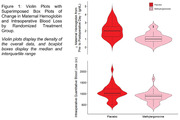

## 40 | Management of Postpartum Preeclampsia and Hypertensive Disorders (MOPP): Postpartum Tight Versus Standard Blood Pressure Control

Emily B. Rosenfeld^1^; Deepika Sagaram^1^; Rachel Lee^1^; Ernani Sadural^2^; Richard C. Miller^2^; Ruby Lin^1^; Deshae Jenkins^1^; Kristin Blackledge^3^; Ivana Nikodijevic^4^; Alex Rizzo^2^; Vanessa Martinez^1^; Emily E. Daggett^5^; Olivia McGeough^1^; Cande V. Ananth^1^; Todd J. Rosen^1^

*^1^Rutgers Robert Wood Johnson Medical School, New Brunswick, NJ; ^2^Cooperman Barnabas Medical Center, RWJBarnabas Health, Livingston, NJ; ^3^Rutgers New Jersey Medical School and Cooperman Barnabas Medical Center, Livingston, NJ; ^4^New Jersey Medical School, Newark, NJ; ^5^Rutgers Robert Wood Johnson Medical School, Edison, NJ*

8:15 AM ‐ 8:30 AM

**Objective**: To assess the effect of lowering the blood pressure (BP) threshold for treatment to 130/80 mmHg from the ACOG standard of 150/100 mmHg, coupled with remote BP monitoring, in managing postpartum patients with hypertensive disorders and reducing Emergency Department (ED) visits.

**Study Design**: A prospective cohort of postpartum patients was recruited in a multicenter study between March 2023 and March 2024 and treated to maintain BP < 130/80 mmHg for six weeks postpartum. These patients were compared to a retrospective cohort of patients delivered from February 2021 to February 2023 who were treated to maintain BP < 150/100 mmHg. Eligible patients were 18 years or older with a diagnosis of hypertensive disorder. The primary outcome was an ED visit for hypertensive disorders. Secondary outcomes included hospital readmissions and changes in BP at six weeks postpartum. We matched subjects between the interventional and the retrospective cohort through the propensity score method.

**Results**: There were 392 patients enrolled in the interventional cohort and 1,204 patients in the retrospective cohort. After the propensity score match, 276 and 429 patients remained in the intervention and retrospective cohorts. ED visits for hypertensive disorders occurred in 3.6% (n = 10) in the interventional cohort and 8.5% (n = 36) in the retrospective cohort (risk difference [RD] ‐4.8, 95% confidence interval [CI] ‐8.2, ‐1.3; matched odds ratio [OR] 0.32, 95% CI 0.10, 1.01). There were fewer hospital readmissions for hypertensive disorders (RD ‐3.2, 95% CI ‐5.7, ‐0.8; OR 0.09, 95% CI 0.003, 2.31). Compared to the retrospective group, systolic and diastolic BP at six weeks postpartum in the intervention group were, on average, 4.4 mmHg (95% CI ‐6.8, ‐2.0) and 3.1 mmHg (95% CI ‐4.9, ‐1.2) lower (**Figure 1**). BP and mean arterial pressure were lower throughout the six weeks in the intervention group (**Figure 2**).

**Conclusion**: Tighter BP control with remote patient monitoring was associated with a clinically meaningful reduction in postpartum ED visits for hypertensive disorders and lower blood pressure at six weeks postpartum.

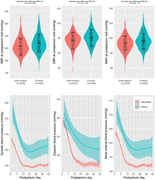

## 41 | Impact of Living in a Food Desert on Prenatal Macro‐ and Micronutrient Intake

Noor K. Al‐Shibli^1^; Anne L. L. Dunlop^2^; Suchitra Chandrasekaran^3^

*^1^Emory University, School of Medicine, Atlanta, GA; ^2^Emory University School of Medicine, Atlanta, GA; ^3^Emory University, Atlanta, GA*

8:30 AM ‐ 8:45 AM

**Objective**: Nutritional deficiencies and associated adverse pregnancy outcomes are emerging threats to public health. Little is known about the impact of the lived food environment on prenatal diet quality. This study aimed to explore the impact of living in a food desert (FD) on macro‐ and micro‐nutrient intake in pregnancy.

**Study Design**: We performed a single‐center prospective pregnancy cohort study from 2014‐2024. Participants were a subset of those enrolled in the Atlanta African American Maternal‐Child Cohort, which included pregnant people ages 18‐40 with a singleton 8‐14 weeks’ gestation who identified as Black/African American. Block‐Bodnar food frequency questionnaires (FFQ) were administered at 8‐14 or 24‐30 weeks’ gestation.  Responses were used to estimate dietary intake of macro/micronutrients. Address of primary residence was assigned to a census tract; FD status was assigned to low‐income & low‐access tracts according to U.S. Department of Agriculture (USDA) definitions. Descriptive and bivariate statistics were utilized.

**Results**: Of n = 543 pregnancies, n = 242 (45%) lived in a FD. Median BMI was 28.3 and 27.1 in FD and non‐FD, respectively (p = 0.321). FD census tracts had more housing units that utilized Supplemental Nutrition Assistance Program (SNAP) benefits: 586 vs 362 (p< 0.0001). Living in a FD was not associated with significant differences in macro/micronutrient intake (Table 1). However, a large proportion of FD & non‐FD cohorts had insufficient micronutrient intake. Rates of insufficient intake were highest for vitamin D, magnesium, folic acid, & iron (Table 2).

**Conclusion**: Interestingly, our data demonstrates no difference in prenatal intake of macro/micronutrients according to residence in a FD. Both groups had high rates of insufficient micronutrient intake regardless of living in a FD or non‐FD. These findings highlight the current prenatal nutritional crisis and support a shift in policy focus to nutrition education, food prescription programs, and diet culture to optimize maternal nutrition status in pregnancy and beyond.

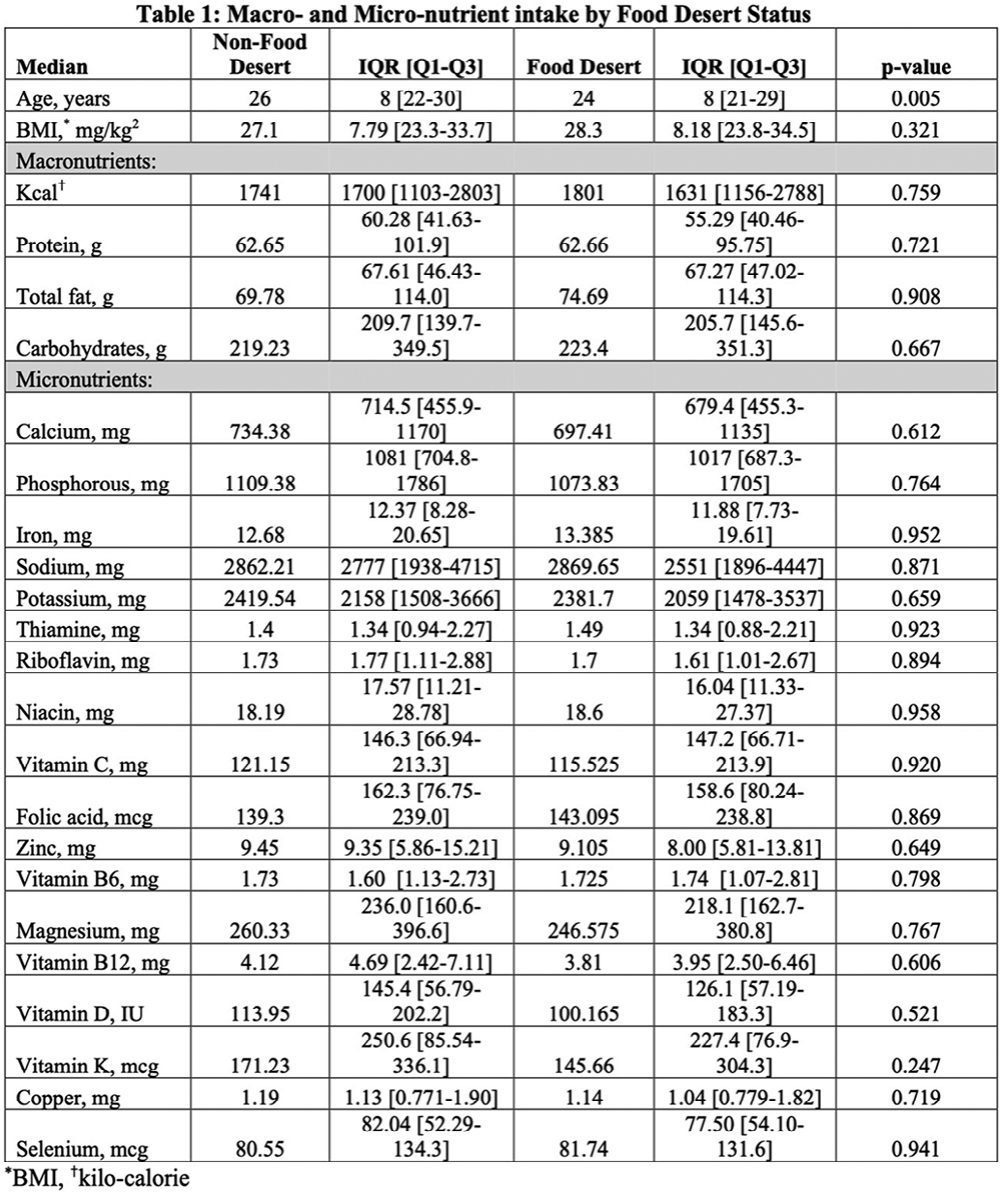

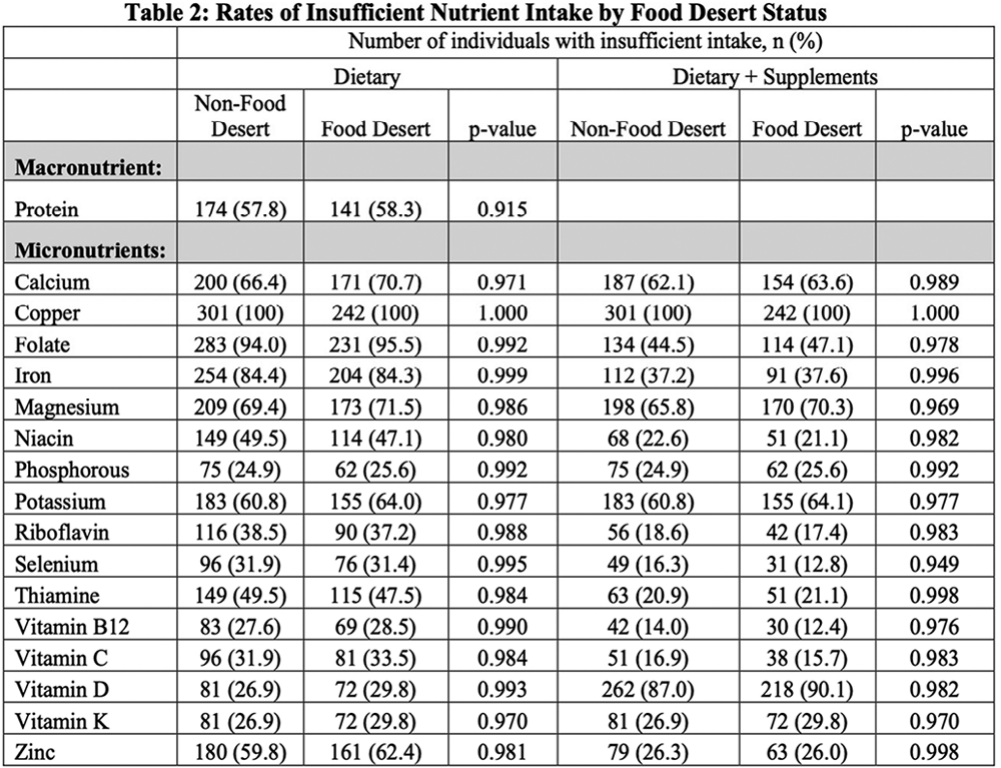

## 42 | Small for Gestational Age Prediction Using Unsupervised Machine Learning of First Trimester Fetal Cardiac Parameters

Rebecca Horgan^1^; Elena Sinkovskaya^1^; Erkan Kalafat^2^; George R. Saade^1^; Alfred Abuhamad^3^

*^1^Macon & Joan Brock Virginia Health Sciences Eastern Virginia Medical School at Old Dominion University, Norfolk, VA; ^2^Koc University Hospital, Istanbul, Istanbul; ^3^Eastern Virginia Medical School, Norfolk, VA*

8:45 AM ‐ 9:00 AM

**Objective**: To use unsupervised machine learning techniques on first trimester fetal cardiac parameters to predict risk of subsequent small for gestational age (SGA) birthweight.

**Study Design**: This was a prospective cohort study which enrolled patients at ≤ 13+6 weeks’ gestation without fetal, umbilical cord or placental abnormalities. At the 1st trimester ultrasound, the chest area, heart area, ventricular inlet lengths, spectral and color Doppler of the atrioventricular valves, valvular regurgitation and myocardial performance index were assessed. K‐Medoids clustering was applied to sort fetuses into risk groups for SGA, defined as a birthweight < 10^th^ percentile for gestational age. Candidate variables were selected with regression analyses and the Elbow method was used to determine the optimal number of clusters. Cumulative rates of outcomes were plotted with Kaplan‐Meier analysis and model performance was tested with area under the curve values with repeated cross‐validation.

**Results**: 617 pregnancies were included in the analysis, of whom 45 (7.3%) delivered an SGA neonate. Z‐scores of chest area (P = 0.027) and tricuspid valve E/A ratio (P< 0.001) showed an independent association with delivery of an SGA neonate and were used in the clustering algorithm. Unsupervised learning algorithm found 3 risk clusters, low (n = 202), intermediate (n = 217), and high (n = 198). The rates of SGA (1.0%, 5.5% and 15.7%, P< 0.001) and non‐reassuring fetal heart rate tracings (3.0%, 6.0% and 9.1%, P = 0.036) differed significantly between the three risk clusters (Figure 1). AUC values of the model in cross‐validation samples were 0.79 (IQR: 0.76–0.81). Using low risk cluster as a threshold, model sensitivity was 95.5% and specificity was 35.0%. The negative predictive value for ruling out SGA was 99.0%.

**Conclusion**: Unsupervised machine learning of first trimester fetal cardiac parameters can effectively stratify risk for SGA neonates. Our findings support the hypothesis that fetal growth trajectory is impacted by early development and may explain the association between fetal growth and programming of adult cardiovascular function.

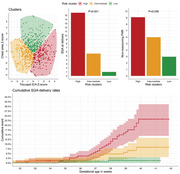

## 43 | Pravastatin Dose‐Range and Pharmacokinetic Study in a Pregnant Rat Model

Xiao‐Yu Wang^1^; Sydney Lammers^2^; Jennifer Reno‐Graber^3^; Frederick Reno^3^; Alan Hoberman^4^; George R. Saade^5^; Monica Longo^6^; Victoria L. Pemberton^7^; Ronald J. Wapner^8^; Nicole Abbott^1^; Zhiliang Xie^1^; Joo Young Na^1^; Mitch A. Phelps^1^; Maged M. Costantine^1^

*^1^The Ohio State University, Columbus, OH; ^2^The Ohio State University, College of Medicine, Columbus, OH; ^3^Reno & Associates, FL; ^4^Charles River Laboratories, PA; ^5^Macon & Joan Brock Virginia Health Sciences Eastern Virginia Medical School at Old Dominion University, Norfolk, VA; ^6^National Institute of Child Health and Human Development, Bethesda, MD; ^7^National Heart, Lung, and Blood Institute, Bethesda, MD; ^8^Columbia University, New York, NY*

9:00 AM ‐ 9:15 AM

**Objective**: Pravastatin has biologic plausibility and promising preliminary clinical data for the prevention of preeclampsia. We sought to determine its pharmacokinetic (PK) properties and biodisposition in a Sprague Dawley (SD) pregnant and juvenile offspring rat model.

**Study Design**: Pregnant female SD rat dams (n = 18/dose) were administered pravastatin by gavage at doses of 0 (ctrl), 15, 45, and 90 mg/kg/day starting on gestation day (GD) 6 through postnatal day (PND) 6. Randomly selected male and female juvenile pups were dosed at 0 (ctrl), 15, 45 and 90 mg/kg/day (n = 18/sex/dose) on PND7 until PND21. Brain, liver, and plasma samples were collected, and pravastatin and its primary metabolite, 3α‐hydroxy pravastatin, were measured with validated HPLC assay to calculate PK parameters on GD20 F0 dams, GD20 F1 fetuses (pooled), and PND21 F1 male and female pups.

**Results**: Pravastatin at all doses was well tolerated in the F0 dams and F1 pups and did not affect growth or development of the F1 pups. Incremental dosing was associated with increasing plasma exposures in F0 dams, F1 fetuses, and F1 pups (AUC and Cmax; Tables 1, 2). Exposure levels in the dams was equivalent to 5‐ to 67‐fold increase over those observed in pregnant women on pravastatin 20mg/day (Table 1). GD20 PK data show very low transfer of pravastatin from dams to fetuses, with F1 fetus exposures ∼≤5% that of F0 dams (Table 1). Direct dosing of F1 pups resulted in systemic exposure that was significantly higher than exposure of the dams. Brain PK data show very low brain exposure for both pravastatin and 3α‐hydroxy pravastatin in F1 GD20 fetuses and PND21 pups (Tables 1, 2). Liver PK profiles show dose‐dependent increased exposure compared with brain at similar doses.

**Conclusion**: In a rat model, maternal to fetal transfer of pravastatin is minimal and fetal and postnatal juvenile pup brain exposure to pravastatin and its metabolite is limited. Our studies also demonstrate no overt toxicity in the dams or offspring with antenatal pravastatin use and support the continued research in repurposing of pravastatin for preeclampsia prevention.

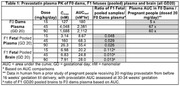

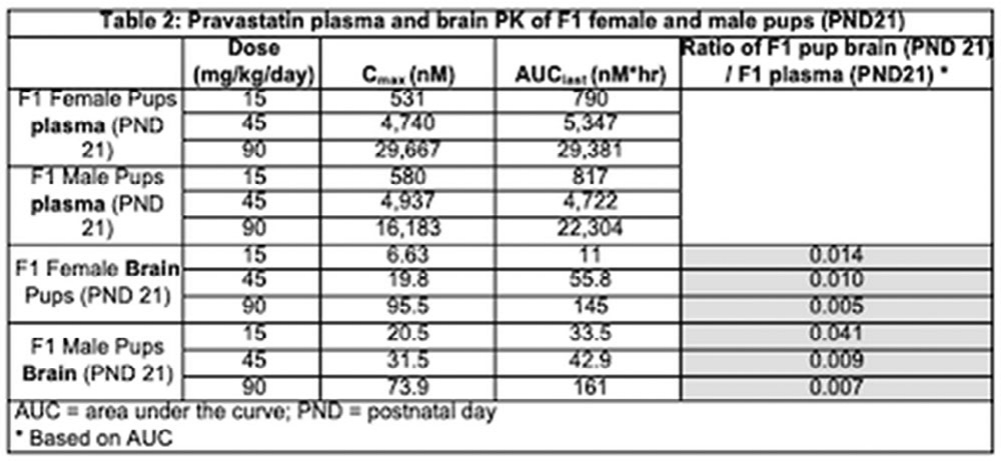

## 44 | Patterns of Perceived and Everyday Stress, Resilience, and Adverse Placentally Mediated Outcomes

Maura Jones Pullins; Sarah Heerboth; Joy McNeal; Ashlyn Tolbert; Annie Dude; Johanna Quist‐Nelson; Rebecca Fry; Tracy A. Manuck

*University of North Carolina, Chapel Hill, NC*

9:15 AM ‐ 9:30 AM

**Objective**: Prenatal stress is common. However, the quantity and type of stress throughout pregnancy remains poorly defined, and the magnitude of the association between stress, resilience, and hypertensive disorders of pregnancy (HDOP) remains poorly understood.

**Study Design**: Primary analysis of 2 prospective cohorts, 2017‐2022. Patients were recruited < 28 weeks’ and completed the Perceived Stress Scale (PSS, range 0‐40; ‘high’ stress is ≥ 21), Everyday Stress Index (ESI, range 0‐60; higher values = higher stress), and the Brief Resilience Scale (BRS, range 1‐5; ‘low’ resilience is < 3) surveys. The primary outcome was plac‐comps defined as hypertensive disorders of pregnancy ± birthweight < 10% for GA and sex, ± placental abruption). We evaluated whether survey scores in early pregnancy (< 24 weeks) were associated with the eventual development of plac‐comps. Linear regression was used to evaluate whether survey results < 24 weeks assessing stress (by PSS, ESI, or both) and/or resilience were associated with the eventual development of plac‐comps.

**Results**: 1,133 individuals met inclusion criteria; 267 (24%) had plac‐comps. Cohort characteristics are shown in Table 1. In general, stress, as quantified by the PSS and ESI, tended to decrease with increasing gestational age, while resilience increased (Figure 1). Overall scores were similar for those with and without plac‐comps. However, when considering survey results < 24 weeks, PSS scores were higher and BRS lower among those who developed plac‐comps compared to those who did not. In regression models controlling for CHTN, pre‐gestational DM, pre‐pregnancy BMI, maternal race, and history of PTB, increased resilience (quantified by BRS < 24 weeks) was associated with a reduced odds of plac‐comps (aOR 0.73, 95% CI 0.56, 0.95). Neither stress score was associated with plac‐comps in regression models.

**Conclusion**: Overall, stress decreases and resilience increases throughout gestation. Increased resilience in early pregnancy is associated with reduced placentally‐mediated adverse outcomes.

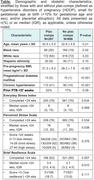

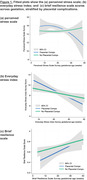

## 45 | Microfluidic Device Successfully Replaces Traditional Models of Pregnancy Associated Drug Pharmacokinetic Studies

Ana Collins‐Smith; Ananth Kammala; Lauren Richardson; Xiao‐ming Wang; Ramkumar Menon

*University of Texas Medical Branch, Galveston, TX*

9:30 AM ‐ 9:45 AM

**Objective**: Pregnant & lactating people remain therapeutic orphans. To mediate this problem, we evaluated drug propagation using a microphysiologic (MPS) model of the human maternal‐fetal interface (FMi‐PLA‐OOC) with simulation software to see if this could serve as an alternative & more efficient approach to studying pharmacokinetics across the placental barrier during pregnancy surpassing the limitations of traditional placental perfusion systems & animal models.

**Study Design**: The humanized FMi‐PLA‐OOC is composed of 7‐cell culture chambers & connected by microchannels containing seven different cells from the feto‐maternal interface. A physiological dose of indomethacin (15µg/mL) was introduced into the decidual chamber to assess drug pharmacokinetics. Media & supernatants were collected at various time points & analyzed using mass spectrometry. A physiologically based pharmacokinetic (PBPK) pregnancy model was developed using Gastroplus & compared with placental perfusion studies, animal models, and clinical data from the literature.

**Results**: Cells in the FMi‐PLA‐OOC maintained viability, metabolism, & did not show cytotoxicity. The drug propagated & reached the placental layers in 4 hours and the fetal membrane cellular layers in 1 hour. Comparing maximum drug concentrations ratios in the fetus and mother (Cmax, fetal/Cmax, maternal) amongst the different platforms demonstrated: 0.45 (placental perfusion), 0.97 (pregnant humans), 0.77 (FMi‐PLA‐OOC), 0.88 (PBPK). The fold error of FMi‐PLA‐OOC & PBPK for Cmax ratios compared to human studies were 0.8 and 0.9, demonstrating an acceptable model.

**Conclusion**: The utilization of the innovative feto‐maternal interface MPS model & PBPK simulation yielded data comparable to the current traditional & simulation approaches. Our humanized MPS model can determine other pharmacologic parameters (efficacy, toxicity, metabolism, absorption, excretion), making preclinical trials easier, cheaper, and faster and in compliance with the FDA Modernization Act 2.0.

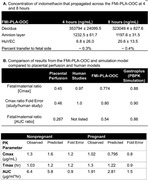

## 46 | Continuous Glucose Monitoring for Gestational Diabetes Diagnosis: A Comparative Effectiveness Randomized Control Trial (PRECISE)

Sarah A. Nazeer^1^; Joycelyn A. Corntwaithe, RD, CDE^1^; Rafael Bravo Santos^1^; Claudia Pedroza^1^; Sean C. Blackwell^2^; Suneet Chauhan^3^; Farah H. Amro^2^; Ghamar Bitar^2^; Jon Tyson^1^; Baha M. Sibai^2^; Michal Fishel Bartal^4^

*^1^The University of Texas Health Science Center at Houston (UTHealth), Houston, TX; ^2^McGovern Medical School at UTHealth Houston, Houston, TX; ^3^Christiana Care, Newark, DE; ^4^UTH Houston & Sheba Medical Center Israel, Houston, TX*

9:45 AM ‐ 10:00 AM

**Objective**: Continuous glucose monitoring (CGM) offers innovative strategies to improve outcomes for gestational diabetes (GDM). We aimed to test the utility of new technology in the diagnosis of GDM compared to the 2‐step method.

**Study Design**: Individuals with a singleton pregnancy, at 24‐30 weeks gestation were randomly allocated to screening with either CGM (Dexcom G6) for 7 days or 2‐step method after stratifying by BMI (≤ 40) and site (03/2023‐04/2024). Individuals diagnosed with fetal anomalies or pregestational DM were excluded. Diagnosis of GDM was made by CGM if any of the following: Time above range (TAR) (≥140 mg/dL) ≥ 10%, mean glucose ≥ 130, or any glucose ≥ 200. GDM was diagnosed by 3‐hour GTT for the routine group. Following GDM diagnosis, both groups received usual care. Primary outcome was a composite of adverse neonatal outcomes (LGA, shoulder dystocia, birth injury, need for IV/PO glucose, respiratory distress, and fetal/neonatal death).  We utilized Bayesian statistics to estimate a sample size of 814 to have a posterior probability of 75% of any reduction of the primary outcome, assuming 90% power with a 30% reduction with CGM. Analysis was done with an intention‐to‐treat under a Bayesian framework with a neutral informative prior and with frequentist statistical analysis (NCT05430204).

**Results**: A total of 814 participants were randomized: 405 in the CGM group, and 409 in the 2‐step method group. Groups were similar at baseline (Table 1). Primary outcome was unavailable for 34 (4.2%).  GDM was diagnosed in 89 (22%) in the CGM group and 63 (15%) in the 2‐step group. Primary neonatal outcome was similar between the groups (34% v. 29%; RR 1.13, 95% Credible interval 0.93‐1.4, posterior probability 88%, Table 2). There were no differences in secondary outcomes. In a prespecified, secondary analysis, no difference in primary outcome among individuals treated for GDM was found (50% v. 37%; RR 1.13 95% CI 0.96‐1.32).

**Conclusion**: In this RCT, the use of CGM for diagnosis of GDM was associated with an increased rate of GDM and similar rates of adverse outcomes compared to the 2‐step method.